# The Retrieval Practice Hypothesis in Research on Learning by Teaching: Current Status and Challenges

**DOI:** 10.3389/fpsyg.2022.842668

**Published:** 2022-05-11

**Authors:** Keiichi Kobayashi

**Affiliations:** Faculty of Education, Shizuoka University, Shizuoka, Japan

**Keywords:** learning by teaching, explaining to others, retrieval practice, retrieval-based learning, student learning

## Abstract

To explain why students learn effectively by teaching, explaining to others in particular, Koh and colleagues advanced the retrieval practice hypothesis, which attributes the learning benefits entirely to the effect of practicing retrieval, that is, effortfully recalling to-be-taught information for the provision of instructional explanations. After delineating the rationale behind the retrieval practice hypothesis, the current situation of research, and the limitations of the existing approach, this paper proposes three tests for the evaluation of the hypothesis that address (1) whether explaining to others after initial studying surpasses restudying in learning performance, (2) whether the amount of effort to retrieve to-be-taught information from memory moderates the learning effects of explaining to others, and (3) whether the degree of elaboration during retrieval practice positively predicts the outcomes of learning by merely recalling to-be-taught information. Evidence is examined regarding whether each test is passed, and future directions for research on the retrieval practice hypothesis are discussed.

## Introduction

Teaching others has been shown to foster one’s learning (Allen, 1976; Annis, 1983; Ehly et al., 1987; Kobayashi, 2019a; Wang et al., 2021). In recent years, a growing body of evidence indicates that students learn effectively even by merely providing others with instructional explanations (Coleman et al., 1997; Rittle-Johnson et al., 2008; Fiorella and Mayer, 2013, 2014; Hoogerheide et al., 2014, 2016, 2019a,b; Koh et al., 2018; Fiorella and Kuhlmann, 2020; Jacob et al., 2020, 2022), except for some cases (Roscoe and Chi, 2008; Lachner et al., 2020, 2021b; Jacob et al., 2021; for meta-analytic reviews, see Kobayashi, 2019b; Lachner et al., 2021b). The provision of instructional explanations includes explaining the contents of learning material to another student face to face (e.g., Coleman et al., 1997), creating an instructional video (e.g., Fiorella and Mayer, 2013; Hoogerheide et al., 2014), and preparing written explanations for a fictitious student’s learning (e.g., Hoogerheide et al., 2016; Lachner et al., 2021b). Unlike explaining to oneself, which is a self-oriented activity, explaining to others requires considering the others’ perspectives and designing explanations for their learning (Wittwer and Renkl, 2008; Chi, 2021). The two types of explaining are also distinguished from one from the other in that they have different effects on learning (Coleman et al., 1997; Rittle-Johnson et al., 2008; Roscoe and Chi, 2008; Lachner et al., 2021b).

Several theoretical accounts have been offered regarding when and why explaining to others fosters one’s learning. For example, Fiorella and Mayer’s (2013, 2014) generative learning hypothesis argues that the generation of instructional explanations stimulates generative processing, that is, selecting to-be-taught information from learning material, organizing the selected information meaningfully, and integrating it with prior knowledge, which lead to successful learning. Roscoe and Chi (2007) and Lachner et al. (2020) emphasize the role of metacognitive processes as well, proposing that students benefit from explaining to others if it acts as a trigger for self-monitoring of comprehension, thereby promoting constructive or generative processing. The social presence hypothesis (Hoogerheide et al., 2016; Lachner et al., 2021a) holds that the level of social presence—the degree to which one’s student is perceived realistically in a mediated communication environment—determines the learning effects of explaining to others. Among other accounts, this paper focuses on the retrieval practice hypothesis, which was advanced by Koh et al. (2018) as an alternative to the existing accounts and has received research attention since.

According to Koh et al.’s (2018) retrieval practice hypothesis, the learning benefits of explaining to others are entirely attributable to the retrieval practice effect. The *retrieval practice effect*, also known as the *testing effect* or *test-enhanced learning*, refers to the enhancement of learning by effortfully retrieving to-be-learned information from long-term memory (e.g., Roediger and Karpicke, 2006). In the typical paradigm of retrieval practice research, students study learning material, practice retrieval (e.g., recall the studied contents) after a short or long delay, and finally take a criterion test (Karpicke, 2017). Retrieval practice is induced not only by free-recall, cued-recall, or recognition testing (Roediger and Karpicke, 2006; Rowland, 2014) but also by engaging in a retrieval-based learning activity, for example, creating a concept map in a closed-book style (Blunt and Karpicke, 2014; Karpicke et al., 2014a) and explaining from memory (Hinze et al., 2013; Hiller et al., 2020). There is considerable evidence that retrieval practice surpasses restudying or other learning strategies in criterion test performance, such as retention, inference, and transfer (for meta-analytic reviews, see Rowland, 2014; Adesope et al., 2017; Pan and Rickard, 2018; Yang et al., 2021), though, under certain conditions, retrieval-based learning has been shown to be inferior to non-retrieval-based learning, such as generative learning strategies (e.g., Roelle and Berthold, 2017; Roelle and Nückles, 2019; Ebersbach, 2020; Hiller et al., 2020). Recent research on learning by teaching has often employed an experimental paradigm consisting of three phases (e.g., Fiorella and Mayer, 2013; Hoogerheide et al., 2014). In the first phase, students study learning material with or without the expectation of teaching. After a short while, in the second phase, they provide instructional explanations of the studied contents without referring to the learning material or their notes. In the third phase, their learning outcomes are assessed. This paradigm appears to fulfill the basic requirements for retrieval-based learning. That is, students must recall to-be-taught information with some effort for the provision of instructional explanations before they take a criterion test. Thus, the retrieval practice hypothesis posits that retrieval practice accounts for the effectiveness of learning by explaining to others. Although promising, no attempt has been made to summarize and carefully examine evidence regarding this hypothesis. In this paper, I discuss where research on the retrieval practice hypothesis is and where it should go, pointing out the limitations of the existing approach and proposing informative tests for the evaluation of the hypothesis.

## Current Situation of Research on the Retrieval Practice Hypothesis

To examine the retrieval practice hypothesis, Koh et al. (2018) had students study a text first and then either (a) create an instructional video about the contents of the text without referring to the text or their notes (i.e., practice retrieval while explaining), (b) take a free-recall test on the text (i.e., practice retrieval without explaining), (c) read a given teaching script aloud in the presence of a video camera (i.e., explain without practicing retrieval), or (d) perform a filler task (i.e., spend extra time completing another activity without internal or external re-exposure to the text information) before a final comprehension test. The instructional video and recall test groups performed better in comprehension than the teaching script group (*d*s = 0.57 and 0.65, respectively) and the filler task group (*d*s = 0.82 and 0.91, respectively). The former two groups did not statistically significantly or substantially differ from each other (*d* = 0.10). Koh et al. (2018) concluded from these results that retrieval practice, but not explanation generation, produces the learning benefits of explaining to others.

However, the findings of Koh et al. (2018) are not definitive yet. As Lachner et al. (2020) noted, reading the given teaching script aloud may have excluded not only the retrieval of to-be-taught information but also the generation of instructional explanations. It is questionable whether Koh et al. (2018) successfully separated the retrieval practice and explanation generation effects by comparing the instructional video and teaching script groups. Furthermore, existing evidence is mixed on the learning effects of explaining to others versus recalling. In line with Koh et al.’s (2018) findings, some studies have found no statistically significant differences in learning performance between explaining to others and recalling (Hoogerheide et al., 2016, Experiment 1; Lachner et al., 2020, 2021a; Jacob et al., 2021). Effect sizes reported in or calculated from these studies were *d*s = −0.47–0.28. In contrast, other studies have shown that, under certain conditions, learning by explaining to others outperforms learning by recalling (Hoogerheide et al., 2014, 2016, Experiment 2; Jacob et al., 2020; reported *d*s = 0.36–1.10). The observed superiority of explaining to others over recalling has been interpreted by opponents of the retrieval practice hypothesis to suggest that the retrieval practice effect does not fully account for the learning benefits of explaining to others (Hoogerheide et al., 2014; Lachner et al., 2020).

More importantly and contrary to the assumption underlying previous work on the retrieval practice hypothesis (e.g., Hoogerheide et al., 2014; Koh et al., 2018; Lachner et al., 2020), examining the learning effects of explaining to others versus recalling is not always informative for the evaluation of the hypothesis. The informativeness is dependent on psychological mechanisms of retrieval-based learning. To illustrate, consider two competing accounts of the retrieval practice effect: the elaborative retrieval hypothesis and the episodic context theory. The elaborative retrieval hypothesis (Carpenter, 2009, 2011; Carpenter and Yeung, 2017) holds that the effortful retrieval of to-be-learned information promotes learning through elaborative processing. Elaborative processing refers to linking retrieval cues with semantically relevant information in long-term memory, thereby creating multiple routes from each retrieval cue to target information that facilitate the later retrieval of the target information. It is also assumed that the degree of elaboration varies depending on how to-be-learned information is retrieved and processed further. For example, elaboration may be induced more effectively when retrieval practice is involved in the act of explaining from memory than taking a recall test (Hinze et al., 2013). If the elaborative retrieval hypothesis is correct, the superiority of explaining to others over recalling in learning performance—the larger versus smaller elaboration (= retrieval practice) effects—does not constitute evidence against the retrieval practice hypothesis. Conversely, the episodic context theory (Karpicke et al., 2014b; Lehman et al., 2014) clearly distinguishes the retrieval practice effect from the elaboration effect and does not conflict with the idea that the two effects can coexist in learning by explaining to others. If instead this account is correct, the examination of learning effects of explaining to others (the retrieval practice and elaboration effects) versus recalling (the retrieval practice effect) may yield insights into the retrieval practice hypothesis. Unfortunately, there is no conclusive evidence to prove either of the accounts. Psychological mechanisms behind the retrieval practice effect are still in dispute (Karpicke, 2017; McDermott, 2021). To effectively evaluate the retrieval practice hypothesis, more informative tests are needed.

## Informative Tests for Evaluating the Retrieval Practice Hypothesis

As an alternative to the existing approach, this paper proposes three tests for the evaluation of the retrieval practice hypothesis. The first test asks whether explaining to others after initial studying surpasses restudying in learning performance. The second test concerns the influence of retrieval effort on learning by explaining to others. It asks whether the amount of effort to retrieve to-be-taught information moderates the learning effects. The third test addresses whether the degree of elaboration during retrieval practice positively predicts the outcomes of learning by merely recalling to-be-taught information. The basic assumption underlying these tests is that if learning by explaining to others and recalling are based on the same mechanism, the two processes will be identical in essential respects. The retrieval practice hypothesis may not be substantiated by passing only one of the tests. But still, whether all the three tests are passed will be informative for judging the validity of the hypothesis. The idea behind each test is as follows:

### Superiority of Learning by Explaining to Others Over Restudying

Successfully retrieving to-be-learned information from memory entails (internal) re-exposure to the information. Therefore, it is important for proponents of retrieval-based learning to rule out the possibility that rather than the process of effortful retrieval, the re-exposure accounts for the retrieval practice effect (Roediger and Karpicke, 2006; Karpicke, 2017). One approach to this problem is to ascertain whether practicing retrieval fosters learning more effectively than restudying, which involves (external) re-exposure to to-be-learned information (Karpicke, 2017; McDermott, 2021). Supporting the basic idea of retrieval-based learning, the superiority of retrieval practice over restudying in learning performance has been shown to be robust (Roediger and Karpicke, 2006; Rowland, 2014; Adesope et al., 2017). If retrieval practice produces the learning benefits of explaining to others, students will learn better when they explain to others after initial studying than when they restudy.

### Influence of Retrieval Effort on Learning by Explaining to Others

It has been suggested that the amount of effort to retrieve (or the level of the difficulty of retrieving) to-be-learned information is a determinant of the retrieval practice effect (e.g., Pyc and Rawson, 2009; Endres and Renkl, 2015; Karpicke, 2017). Unless retrieval failure is high or left unremedied, the greater retrieval practice effect is produced when greater retrieval effort is required (Karpicke, 2017). Examples include when fewer retrieval cues are available for the retrieval of target information (Carpenter and DeLosh, 2006), when a retrieval cue is semantically less related to target information (Carpenter, 2009), and when there is a longer interval between initial studying and retrieval practice (Pyc and Rawson, 2009). Similarly, the more effortful retrieval of to-be-taught information in the process of providing instructional explanations will lead to better learning performance if retrieval practice is a key mechanism behind learning by explaining to others. Excessive sacrifice of retrieval success may reduce the influence of retrieval effort, though.

### The Predicting Effect of Elaboration on Learning by Recalling

As noted previously, researchers still disagree on the role of elaboration in retrieval-based learning. Remarkably, though, research on learning by teaching has shown that students who include more elaborations—ideas beyond learning material, such as bridging inferences, examples, and analogies—in their instructional explanations learn better (Roscoe and Chi, 2008; Roscoe, 2014; Lachner et al., 2018; Fiorella et al., 2021; Jacob et al., 2021, 2022; Kobayashi, 2021a,b). For example, Fiorella and Kuhlmann (2020) found that the number of elaborative statements in instructional explanations students generated during the creation of an instructional video was a positive predictor of their learning outcomes. These findings suggest that the elaborative processing of to-be-taught information makes a significant contribution to learning by explaining to others. Therefore, if the learning benefits of explaining to others and recalling are both entirely attributable to the retrieval practice effect, elaboration will be observed to play a role in learning by merely recalling to-be-taught information as well. More specifically, the number of elaborations in recall protocols generated during retrieval practice will positively predict learning outcomes.

## Applying the Informative Tests: Current Evidence and Future Challenges

Does the retrieval practice hypothesis pass the three informative tests? At this point in time, my answer is “No.” In this final section, I examine empirical evidence regarding whether each test is passed and discuss future research needs and directions.

First, only a few studies have examined the learning effects of explaining to others versus restudying. For example, Hoogerheide et al. (2019b) found that students who explained the contents of learning material to a fictitious student performed better in the acquisition of conceptual knowledge than those who restudied the learning material. Similar results are obtained in Fiorella and Kuhlmann (2020). However, these studies have been concerned with the creation of an instructional video, or the provision of oral explanations. The same may not apply to learning by explaining in writing versus restudying. Indeed, it has been shown that the learning benefits of providing instructional explanations (versus recalling or other learning strategies) are observed when the explanations are provided orally but not when they are written (Hoogerheide et al., 2016; for a meta-analytic review, see Lachner et al., 2021b). This poses a serious challenge to the retrieval practice hypothesis. There is no evidence that oral and written modes of retrieval differentially affect the effectiveness of retrieval-based learning versus restudying (Putnam and Roediger, 2013; for a meta-analytic review, see Yang et al., 2021). The retrieval practice hypothesis, as it stands, cannot explain the potential influence of explanation modality on the learning effects of explaining to others versus restudying. Additional work is needed to determine whether explaining to others surpasses restudying in learning performance, regardless of explanation modality.

Second, to my knowledge, there are no data concerning the influence of retrieval effort on learning by explaining to others. Future research should address this gap. For example, it would be interesting to systematically manipulate the external availability of to-be-taught information and thereby examine its influence on learning effects of explaining to others. The extent to which target information is externally available during retrieval practice affects the amount of effort to retrieve the information (Kornell et al., 2015; Hiller et al., 2020; Waldeyer et al., 2020). Students may expend less effort at retrieving to-be-taught information when they can rely on learning material and/or their notes for the provision of instructional explanations (an open-book style) than when they cannot (a closed-book style). The retrieval practice hypothesis predicts that learning by explaining in a closed-book style will outperform learning by explaining in an open-book style if the closed-book explanation does not severely hamper the successful retrieval of to-be-taught information or if retrieval failure is remedied in some way (e.g., by corrective feedback).

Finally, the predicting effect of elaboration on learning by recalling to-be-taught information remains unexamined. Although as mentioned above, not a few studies have compared the learning effects of explaining to others and recalling (e.g., Koh et al., 2018; Lachner et al., 2020; Jacob et al., 2021), none of them have reported any data concerning whether elaboration plays a role in learning by recalling. Endres et al. (2017) found that the degree of elaboration during retrieval practice was a positive predictor of learning from a lecture video. However, this study differs in many ways from the previous work examining the role of elaboration in learning by explaining to others (e.g., Fiorella and Kuhlmann, 2020). Accordingly, their findings are not directly comparable. Subsequent studies should be designed to compare the predicting effects of elaboration on learning by explaining to others and recalling. It would be informative to test whether the number of elaborations in instructional explanations and recall protocols similarly predict learning outcomes in one study.

## Conclusion

In conclusion, research on the retrieval practice hypothesis is still in its infancy. The currently available evidence is inadequate to assess the hypothesis. Further work could advance the field by verifying whether explaining to others surpasses restudying in learning performance, regardless of explanation modality; whether the amount of retrieval effort moderates the learning effects of explaining to others; and whether the degree of elaboration positively predicts the outcomes of learning by merely recalling to be-taught information. At least there is still no direct evidence showing that the retrieval practice hypothesis fails these tests. The validity of the hypothesis will be strengthened if each test is passed. If not, the likelihood is that psychological mechanisms other than or in addition to retrieval practice account for the learning effects of explaining to others. I hope that this paper inspires more research on the retrieval practice hypothesis and advances the theoretical progress of learning by teaching.

## Author Contributions

The author designed the paper, analyzed the literature, and drafted the manuscript.

## Funding

This work was supported by the Japan Society for the Promotion of Science (Grant-in-Aid of Scientific Research (C)/No. 19K03225).

## Conflict of Interest

The author declares that this work was conducted in the absence of any commercial or financial relationships that could be construed as a potential conflict of interest.

## Publisher’s Note

All claims expressed in this article are solely those of the authors and do not necessarily represent those of their affiliated organizations, or those of the publisher, the editors and the reviewers. Any product that may be evaluated in this article, or claim that may be made by its manufacturer, is not guaranteed or endorsed by the publisher.
